# Hippocampal neurogenesis and volume in migrating and wintering semipalmated sandpipers (*Calidris pusilla*)

**DOI:** 10.1371/journal.pone.0179134

**Published:** 2017-06-07

**Authors:** Nara Gyzely de Morais Magalhães, Cristovam Guerreiro Diniz, Daniel Guerreiro Diniz, Ediely Pereira Henrique, Patrick Douglas Corrêa Pereira, Isis Ananda Matos Moraes, Mauro André Damasceno de Melo, David Francis Sherry, Cristovam Wanderley Picanço Diniz

**Affiliations:** 1 Universidade Federal do Pará, Instituto de Ciências Biológicas, Laboratório de Investigações em Neurodegeneração e Infecção no Hospital Universitário João de Barros Barreto, Belém, Pará, Brasil; 2 Instituto Federal de Educação Ciência e Tecnologia do Pará, Campus Bragança, Laboratório de Biologia Molecular e Neuroecologia, Bragança, Pará, Brasil; 3 University of Western Ontario, Department of Psychology Advanced Facility for Avian Research, London, Ontario, Canada; Bowling Green State University, UNITED STATES

## Abstract

Long distance migratory birds find their way by sensing and integrating information from a large number of cues in their environment. These cues are essential to navigate over thousands of kilometers and reach the same breeding, stopover, and wintering sites every year. The semipalmated sandpiper (*Calidris pusilla*) is a long-distance migrant that breeds in the arctic tundra of Canada and Alaska and winters on the northeast coast of South America. Its fall migration includes a 5,300-kilometer nonstop flight over the Atlantic Ocean. The avian hippocampus has been proposed to play a central role in the integration of multisensory spatial information for navigation. Hippocampal neurogenesis may contribute to hippocampal function and a variety of factors including cognitive activity, exercise, enrichment, diet and stress influence neurogenesis in the hippocampus. We quantified hippocampal neurogenesis and volume in adult migrating and wintering semipalmated sandpipers using stereological counts of doublecortin (DCX) immunolabeled immature neurons. We found that birds captured in the coastal region of Bragança, Brazil during the wintering period had more DCX positive neurons and larger volume in the hippocampus than individuals captured in the Bay of Fundy, Canada during fall migration. We also estimate the number of NeuN immunolabeled cells in migrating and wintering birds and found no significant differences between them. These findings suggest that, at this time window, neurogenesis just replaced neurons that might be lost during the transatlantic flight. Our findings also show that in active fall migrating birds, a lower level of adult hippocampal neurogenesis is associated with a smaller hippocampal formation. High levels of adult hippocampal neurogenesis and a larger hippocampal formation found in wintering birds may be late occurring effects of long distance migratory flight or the result of conditions the birds experienced while wintering.

## Introduction

The semipalmated sandpiper is a long-distance migrant that breeds in the arctic and undertakes an annual fall migration to South America. Geolocation tracking of a semipalmated sandpiper that bred on Coats Island in the Canadian arctic showed a six-day, non-stop flight from a stopover site on James Bay to the Orinoco Delta on the border of Venezuela and Guyana, followed by 11 days of further movement to northeastern Brazil where it spent the winter [1]. Its fall migratory path included a 5,300-kilometer flight over the Atlantic Ocean to South America. Approximately 75% of the world population of semipalmated sandpipers make a stopover during fall migration in the Bay of Fundy in Canada. Birds feed at stopovers to increase their fat reserves in order to sustain intense continuous exercise during the following days of non-stop flight [2].

The avian hippocampus plays a central role in spatial ability and spatial memory in birds as shown by single cell recording [3, 4], the effects of hippocampal lesions [5–8] and comparative analyses [6, 9–17]. There is evidence for hippocampal involvement in the spatial and navigational components of migration and homing [11, 18–20]. Adult neurogenesis in the avian hippocampus is specifically associated with migratory behavior [15, 21] and migratory birds have been shown to have better long term memory [22] and better spatial memory [19, 20] than non-migrants. Migrants combine visuospatial learning and memory with other navigational systems [23, 24] including cryptochrome magnetoreception [25, 26] to maintain orientation during flight. Enhanced adult hippocampal neurogenesis is a strong candidate as one of the mechanisms underlying spatial ability and navigation in migrants [15] and there is recent evidence for glial cell involvement as well [16].

Experiments with immediate early genes (IEGs) have showed significant changes in hippocampal activation patterns confirming the hippocampus role in navigation [27–30]. Indeed, their activation seems to be directly related with memory storage and to an increase in the neuronal activity in response to changes in the magnetic field [30–36].

There are many proposed functions for adult hippocampal neurogenesis. It may provide new neurons to encode new memories [37] or promote both forgetting and the acquisition of new memory [38]. Neurogenesis in the hippocampus may play a role in pattern separation, that is, distinguishing among similar events [39] or it may establish a reserve of new neurons that can be drawn on as needed [40]. The excitability of immature neurons may contribute to the remodelling of hippocampal circuits [41]. There are, in addition, many factors that increase adult hippocampal neurogenesis including cognitive activity, environmental enrichment, exercise, diet, stress, gonadal hormones, and aging [42–47].

Because many of these multivariate influences are present in long distance migration we hypothesized that neurogenesis in the hippocampal formation of migrating birds would be higher than that of wintering birds. Indeed, we expect that the negative influence of the extenuating exercise associated with the long distance migratory flight would be less intense than the other positive influences and this would upregulate neurogenesis. To test this hypothesis, we compared the number of new and adult neurons in the hippocampus of semipalmated sandpipers captured during fall migration in August at stopover in the Bay of Fundy, Canada, with that of individuals captured while wintering in the northeastern coastal region of Brazil near Bragança, Pará.

## Material and methods

A total of thirteen individuals were used, eight migrating *C*. *pusilla* (01,02,03,04,05,11,12,13) were collected in August 2012 at the Bay of Fundy, Canada (45°50'19.3" N and 64°31'5.39"W), we used *C*. *pusilla* 01, 02, 03, 04, 05 for DCX positive cell counts and volume estimation and *C*. *pusilla* 01, 11, 12, 03, 13 for NeuN cell counts. Other five wintering individuals (*C*. *pusilla* 06,07,08,09,10) were captured between November and March on Canela Island (four individuals in 2014 and one in 2009), in the tropical coastal zone of northern Brazil (00°47’09.07” S and 46°43’11.29” W) and these were used only for DCX counts and volume.

**Fig 1:**
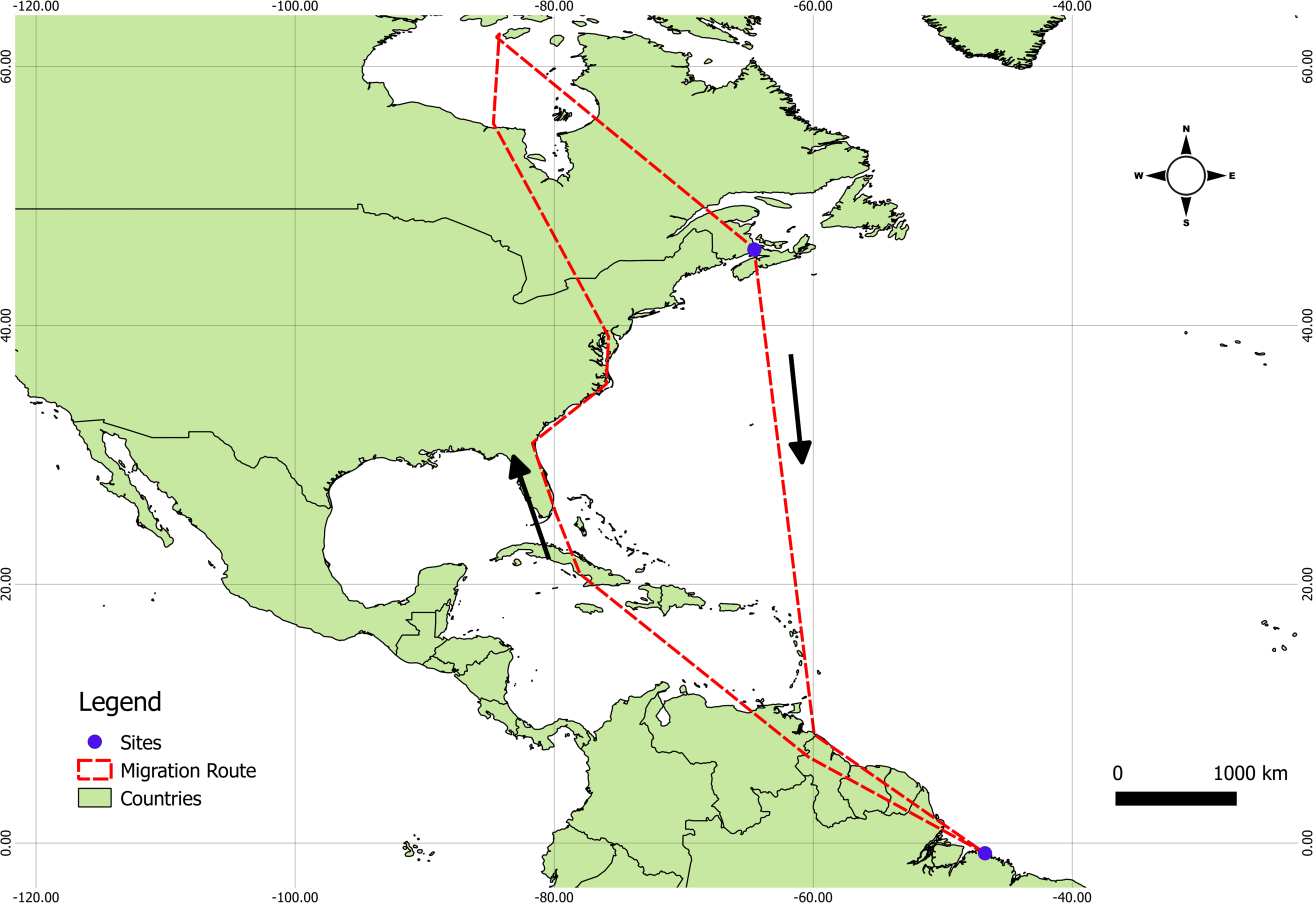
Migratory routes. Map with the migratory routes. Blue dots are the sampling sites. Red traced lines are the routes [48]. Scale bar: 1000 km.

Although the number of birds are different all statistical comparisons, were made between groups of 5 (five) individuals (except for NeuN counts extracted from previous report [16]) assuming unpaired samples and unequal variances. Semipalmated sandpipers reach the coastal zone of northern Brazil in August and September and begin migration to the arctic between May and July (Fig 1).

Birds were captured under license N° 44551–2 from the Chico Mendes Institute for conservation of Biodiversity (ICMBio) and Scientific Capture permit ST2783 from the Canadian Wildlife Service. All procedures were carried out in accordance with National Institutes of Health (USA) and Brazilian regulations for scientific procedures on animals and with approval of the Animal Users Subcommittee of the University of Western Ontario. All efforts were made to minimize the number of animals used and the stress and discomfort to animals.

### Perfusion and histology

Immediately after capture (still on the field) and under deep isoflurane anesthesia, birds were perfused transcardially with phosphate buffered saline 0.1M followed by aldehyde fixatives (4% paraformaldehyde, 0.1 M phosphate buffer, pH 7.2–7.4). Brains were dissected, stored in phosphate buffered saline 0.1M and cut by Vibratome (Leica VT1000S) or freezing microtome (Sliding Microtome, Reichert Jung) in the coronal plane. Six anatomical series of sections (6 parallel series), cut at 60 or 80μm in the freezing microtome or vibratome respectively, were immunolabeled for NeuN or DCX or stained by Nissl. Free-floating sections were immunolabeled with anti-doublecortin antibody (Santa Cruz SC-8066) or NeuN antibody (MiliporeSigma, MAB377), and mounted on glass slides coated with an aqueous solution of gelatin (10%) and chromium potassium sulfate (0.5%). Sections were air-dried at room temperature, dehydrated, cleared in alcohol/xylene series and covered with 50% entellan (Entellan® Novo 107961, Merck Milipore) diluted in xylene and cover slipped.

### Immunohistochemistry

Free-floating sections were pre-treated with 0.2 M boric acid (pH 9) at 70°C for 60 min as an antigen retrieval method, washed in 1% phosphate buffer/saline/triton (PBST) and washed three times for 2 min each time in PBS. Sections were then immersed for 12 hours in 10% Normal Horse Serum Blocking Solution S-2000, Vector Laboratories (for DCX slides) and 10% Normal Goat Serum Blocking Solution S-1000 Vector Laboratories (for NeuN slides) transferred to the primary antibody (Doublecortin C-18, sc-8066 Santa Cruz Biotechnology and Anti-NeuN Mab 377 MilliporeSigma) diluted in PBST 0.3% (1:500) and incubated for 12h at 4°C with gentle and continuous agitation. Washed sections (PBST 1%) were then incubated overnight in the secondary antibody (Biotinylated Horse Anti-Goat IgG Antibody, BA-9500, Vector Laboratories (for DCX) and Biotinylated Goat Anti-Mouse IgG Antibody, BA-9200, Vector Laboratories (for NeuN), 1:400 in PBST 0.3%) followed by 0.3% hydrogen peroxide for 15 minutes, washed three times in PBST for 2 min each time, then immersed in avidin-biotin-peroxidase complex (ABC) solution (Vector Laboratories, Burlingame, CA, USA; 37.5μl A, 37.5μl B in 13.12ml of 0.3% PBST) for 60 minutes. After a 2 min wash in PBS, sections were reacted to visualize DCX immunolabeled neurons using the glucose-oxidase-DAB-nickel method. As a control of the immunohistochemical labeling patterns we omitted the primary antibody and confirmed that the secondary antibody did not produce any unspecific labeling [49].

### Hippocampal and telencephalon volumes

We defined the sandpiper hippocampal formation as comprising the hippocampus proper and the parahippocampal area [16]. To measure hippocampus volumes and the ratio between them we followed the total telencephalon method previously recommended [50]. To that end we used the optical fractionator, a standard stereological method that estimates volumes based on the Cavallieri principle [51]. Values for statistical analyses were extracted from doublecortin labelled anatomical series of sections. The telencephalon (telencephalon + hippocampus) volume was estimated between the first and the last tissue sections of the telencephalon as previously suggested [17].

### Neuronal numbers

**Fig 2:**
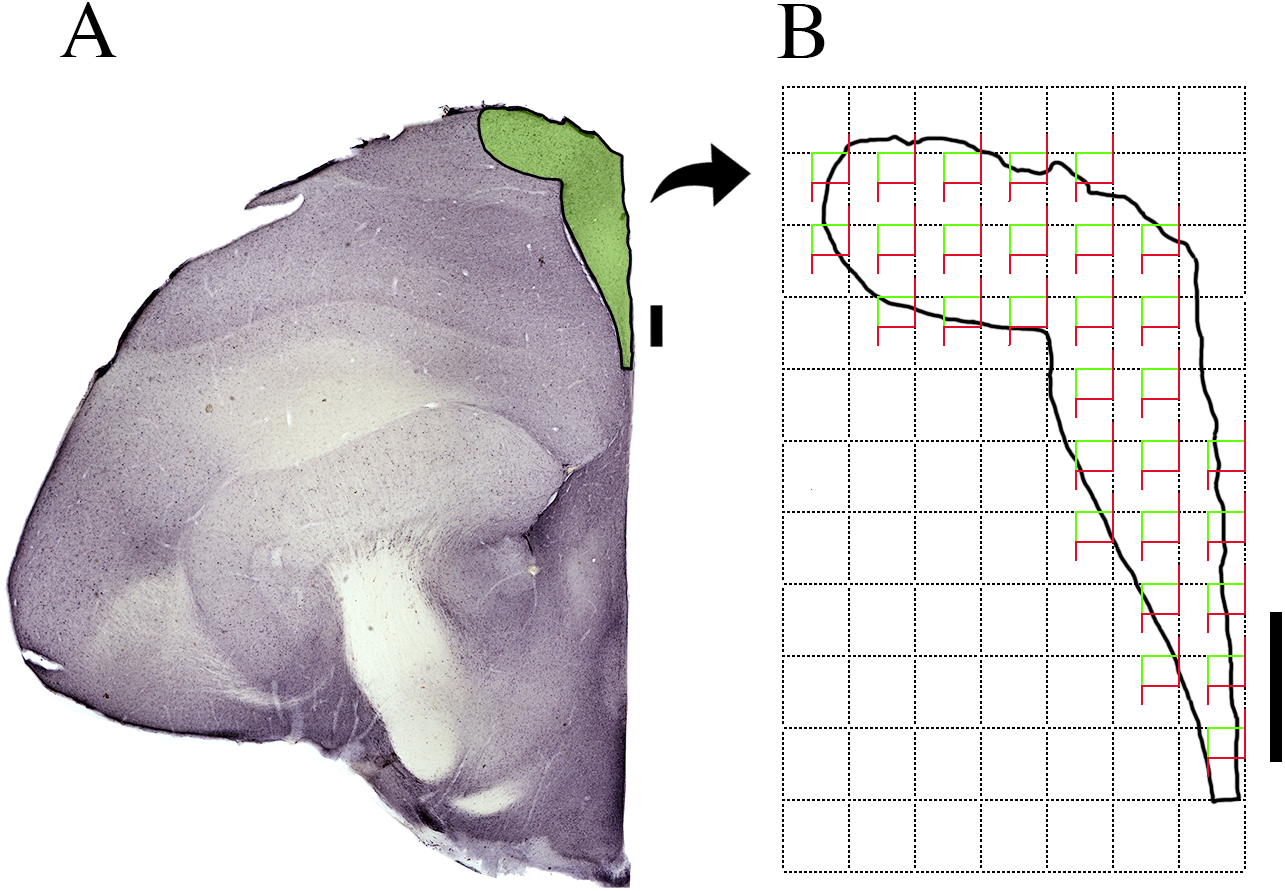
Hippocampal formation grid and counting frames. Example of a section of the left hippocampal formation highlighted in green color (A). Counting frames (140 x 106 μm) were randomly and systematically placed in a 250 x 250 μm grid (B). Scale bars: 500 μm.

After selective DCX and NeuN immunolabeling, we determined the number of neurons. We did not distinguish between migratory (elongate morphology) and recruited (spherical) phenotypes [52, 53]. We used the optical fractionator to estimate total cell numbers [54–56]. The optical fractionator is unaffected by histological changes, shrinkage, or damage-induced expansion of tissue [57]. Each hippocampal contour from one hemisphere was digitized directly from each section using a 4.0X objective on a NIKON Eclipse CI (Nikon, Japan), equipped with a motorized stage (MAC6000, Ludl Electronic Products, Hawthorne, NY, USA). High power images were acquired under oil immersion, using a high-resolution 100x oil immersion plan apo objective (Nikon, NA 1.45, WD = 0.13 μm), and Stereo Investigator software (MBF Bioscience Inc., Frederick, MD, USA). We began by screening the complete section from one hemisphere to delineate the hippocampal region. The borders of the hippocampal formation were deFIned according to the changes identiFIed in the staining pattern. To unambiguously detect and count the objects of interest in the dissector probe, the low power objective was replaced by the high-resolution 100X oil immersion objective. At each counting site, the thickness of the section was carefully assessed using the high-power objective and the FIne focus of the microscope to deFIne the immediate defocus at the top and bottom of the section. Because both the thickness and neuron distribution in the sections were uneven, we estimated the total number of neurons based on the number-weighted section thickness. This value gives the estimated population count determined by the selected series of optical fractionator runs using the number-weighted section thickness [58]. All sampled neurons that came into focus inside the counting frame were counted and added to the total, provided cell bodies were entirely within the counting frame or intersected the acceptance line without touching the rejection line [51]. Counting frames (140 x 106 μm) were randomly and systematically placed in a 250 x 250 μm grid. Fig 2 shows an example of counting frames and grid placed over a section of the left hippocampal formation. The experimental parameters, volumes and counting results in the region of interest of left and right hemispheres are shown for each bird in the supplementary materials Tables A-G in S1 File. The grid size used was adapted to achieve an acceptable coefficient of error (CE). The calculation of the CE for the total neuron count in each bird used in the present study adopted the one-stage systematic sampling procedure (Schaeffer CE) that has been previously validated [59]. The level of acceptable error in the stereological estimations was deFIned by the ratio between the intrinsic error introduced by the methodology and the coefFIcient of variation. CE expresses the accuracy of the cell number estimates, and a CE under 0.05 was deemed appropriate for the present study because variance introduced by the estimation procedure contributes little to the observed group variance [59].

### Photomicrography

**Fig 3:**
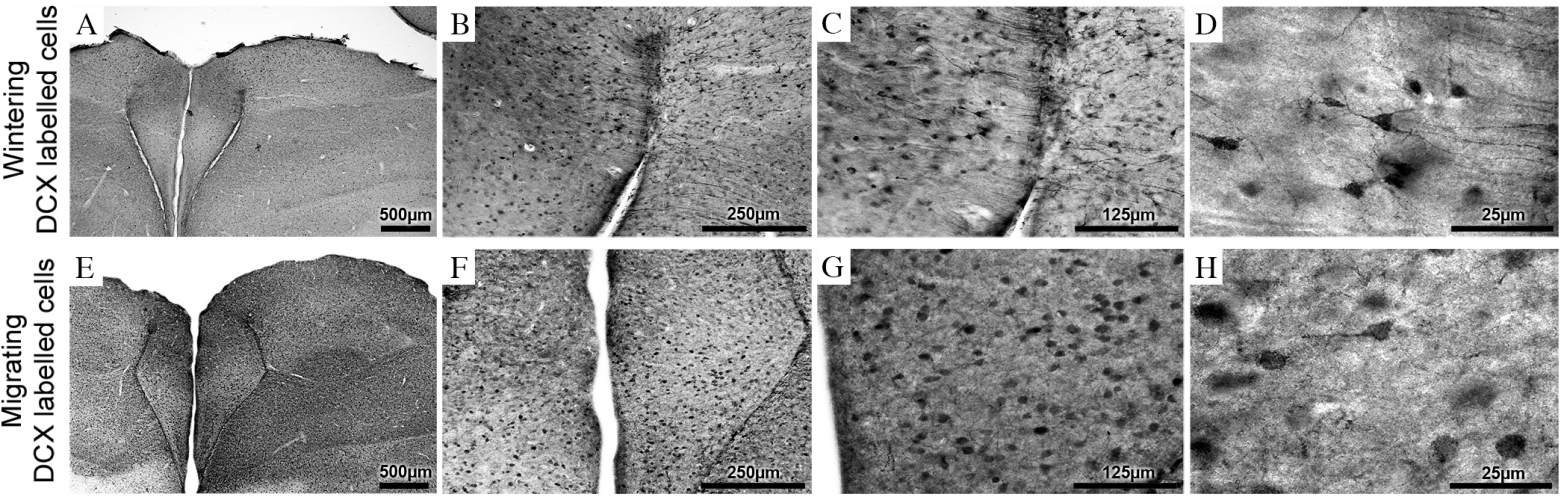
Doublecortin immunolabeled neurons in the hippocampal formation. (A) Wintering hipocampal formation image in 4x objective, (B) 10x objective, (C) 20x objective and (D) 100x objective. (E) Migrating hipocampal formation image in 4x objective, (F) 10x objective, (G) 20x objective and (H) 100x objective. Scale bars: A and E—500 μm; B and F—250μm; C and G—125 μm; D and H—25 μm.

We used a digital camera (Microfire, Optronics, CA, USA) coupled to a NIKON Eclipse Ci microscope to capture digital images which were processed with Adobe Photoshop software for illustrations shown in Figs 2 and 3. Scaling and adjustment of the brightness and contrast levels were applied to the whole image.

## Results and discussion

Doublecortin was broadly expressed in the telencephalon of adult *Calidris pusilla*. Fig 3 shows doublecortin immunolabeled neurons on the hippocampal formation.

**Fig 4:**
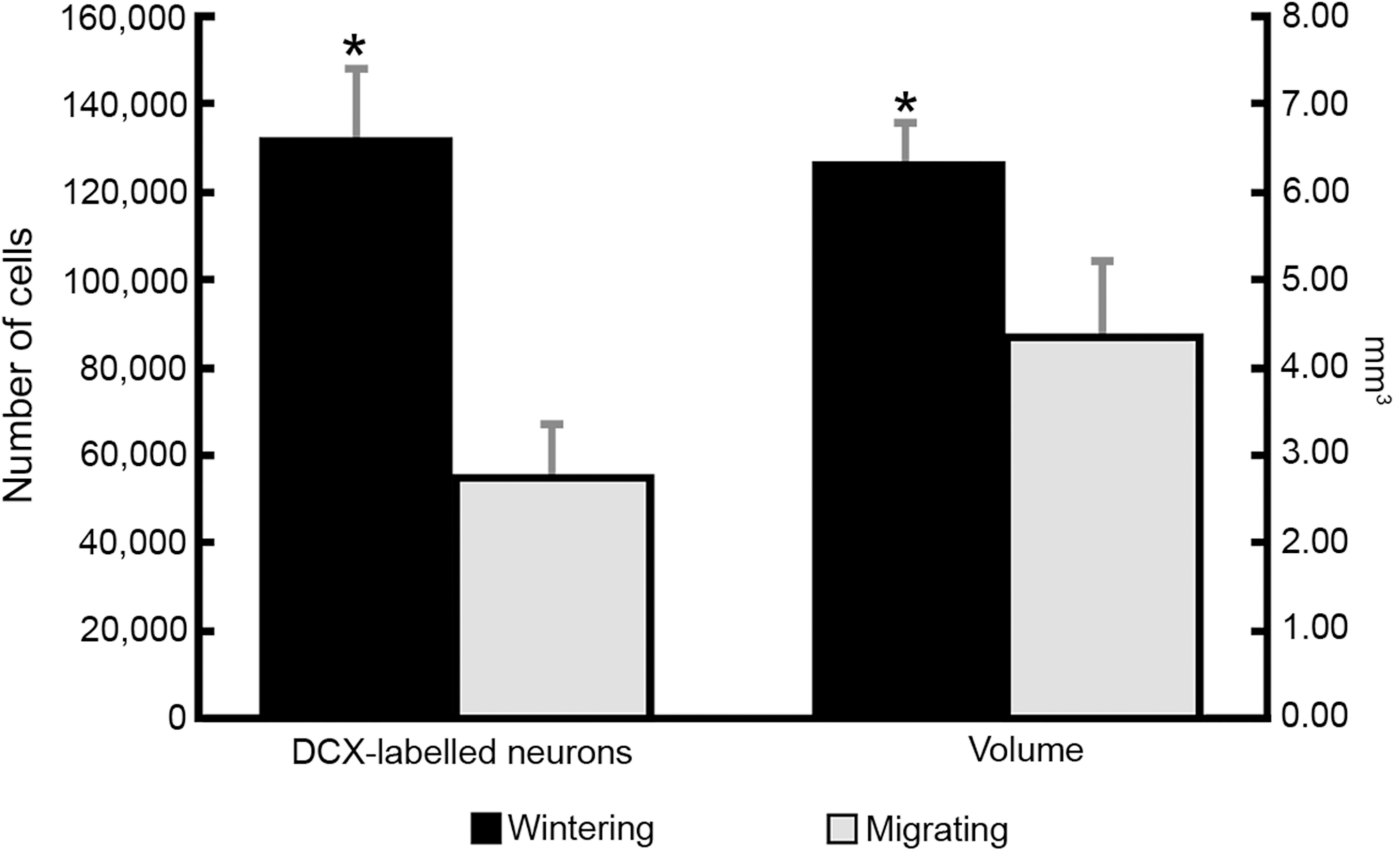
DCX Neurons and volume in migrating versus wintering. Difference between migrating and wintering birds in the number of DCX-immunolabeled hippocampal neurons and hippocampal volume. Asterisks mark significant statistical difference between wintering and migrating animals. Error bars represent standard deviation.

**Fig 5:**
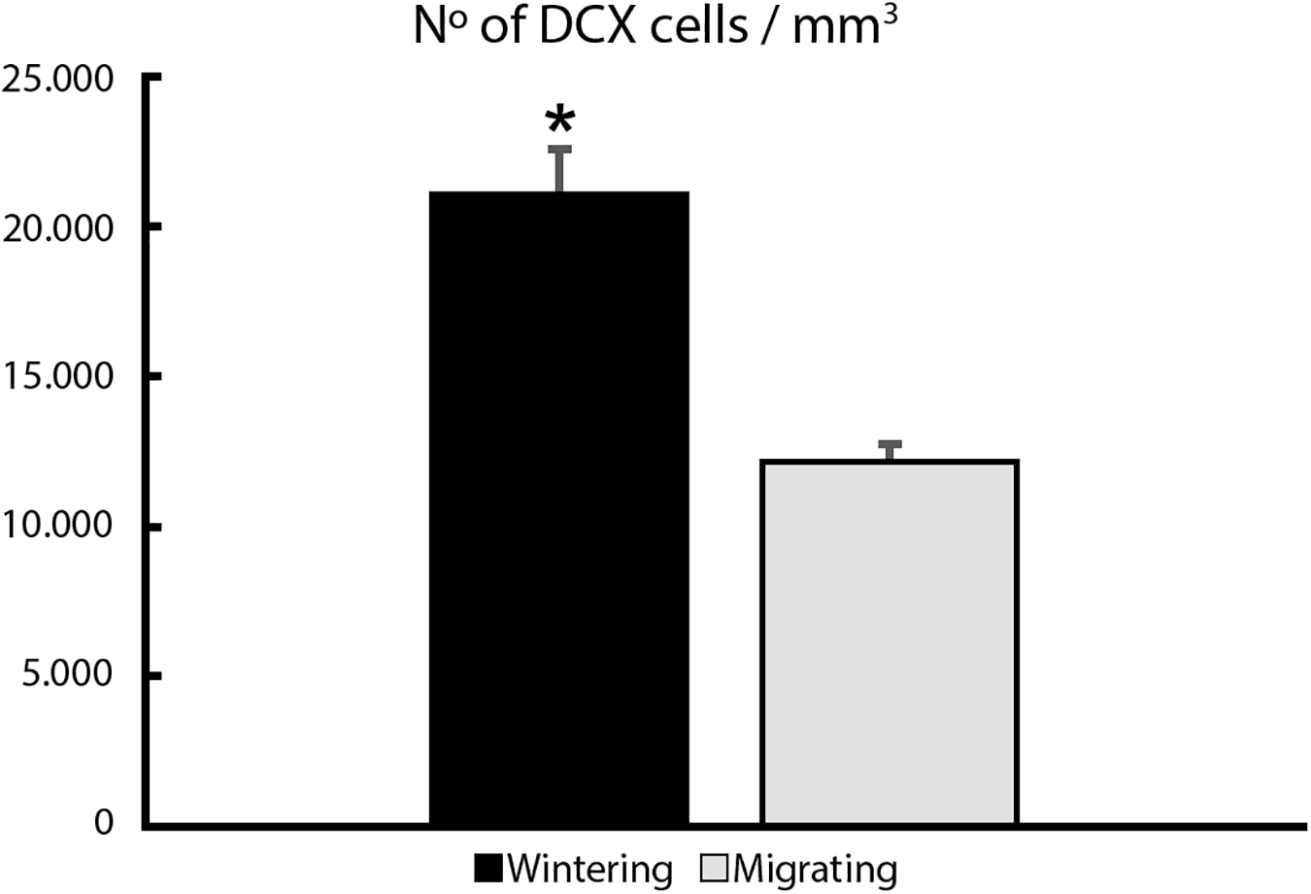
Doublecortin immunolabeled neurons per mm^3^. Difference between migrating and wintering birds in the number of DCX-immunolabeled hippocampal neurons per mm^3^. The asterisk marks significant statistical difference between wintering and migrating animals. Error bars represent standard deviation.

We found a major difference between migrating and wintering birds in the number of DCX-immunolabeled hippocampal cells and in hippocampal volume (Fig 4, Tables E-G in S1 File). *C*. *pusilla* wintering individuals showed on average, 2.4 times more DCX immunolabeled cells than *C*.*pusilla* migrating birds (Wintering 133,143.80 ± 15,551.80 *vs* Migrating 55,057.95 ± 12,171.50 (mean ± SD); two-tailed t-test, df = 7 t = 2.36 p<0.00). As expected the density values of DCX immunolabeled cells showed that wintering birds had 1.73 more DCX positive cells/mm^3^ than migrating ones (Wintering 21,256.95 ± 3,384.1; Migrating 12,257.19 ± 1,065.42 (mean ± SD); two-tailed t-test, df = 7; t = 2.01; p<0.00) (Fig 5). Total number of DCX labelled cells for each bird are shown in Table E is S1 File. The volumes of the hippocampal formation were significantly different in migrating and wintering semipalmated sandpipers (Wintering 6.28 mm^3^ ± 0.3 *vs* Migrating 4.46 mm^3^ ± 0.7 (mean ± SD); two-tailed t-test, df = 5; t = 2.01, p<0.00) shown in Table F in S1 File.

NeuN counts showed on average, no significant differences between migrating and wintering sandpipers. Indeed, NeuN total counts were 946,247 ± 139,352 neurons on migrating and 909,540 ± 138,470 (mean ± SD) on wintering sandpipers (data extracted from [16]) (two-tailed t-test, df = 7; t = 0.39, p = 0.7).

Detailed cell counts and volume data are shown in supplementary material for both hemispheres (Tables A-G in S1 File).

The fall migration of the semipalmated sandpiper includes continental stopover sites and a multi-day nonstop flight across the Atlantic Ocean from northeastern North America to northeastern South America. The environment through which the birds fly changes dramatically during this flight and birds probably integrate global cues, learned local gradient maps, and local landmark information in order to successfully complete migration [24, 60]. Trans-oceanic and trans-continental long-distance navigation makes use of celestial and geomagnetic information [61, 62] whereas learned gradient maps and local landmarks in various sensory modalities are associated with short distance overland migratory behavior [61, 63]. We have shown for the first time in the present report that levels of neurogenesis and volume of the hippocampal formation of a long-distance migrant are lower during fall migration than while wintering.

It is not clear why hippocampal neurogenesis might differ between wintering and actively migrating birds. Cognitive activity, environmental enrichment, diet, and stress are known to affect levels of hippocampal neurogenesis. It is difficult to distinguish their relative contributions to neurogenesis. Indeed, it has been previously described that migration, as noted above, probably engages several cognitive processes. Increased spatial processing is associated with an increase in the number of new neurons in the hippocampus [14, 64–67] and birds’ behavior during migration is consistent with elevated demands on spatial learning and memory [15]. Migratory birds, compared to non-migrants, show better performance in both spatial memory [19, 20] and long term memory tasks [22]. New neurons migrate, enter neural networks, and become important for spatial discrimination [68–71] and spatial memory and learning [14, 72–74]. In line with these results, previous research with migrating (*Zonotrichia leucophrys gambelii*) and non-migrating (*Z*. *l*. *nuttalli*) white-crowned sparrows showed a greater number of doublecortin-positive neurons in the hippocampus of the migratory subspecies [15].

In addition, long distance migration might also be considered a kind of environmental enrichment. The journey may be visuo-spatially enriched and may engage perceptual processes involved in celestial, olfactory and geomagnetic navigation in ways that do not occur outside the period of migration. Long distance migrants may be exposed to more diverse spatial information, compared to short distance migrants, resulting in greater recruitment of new neurons [21]. From mammals, studies in rats and mice showed that environmental enrichment is associated with elevated neurogenesis and neuronal recruitment in the dentate gyrus [75–80].

If increased cognitive activity and environmental enrichment are indeed the causes of increased hippocampal volume and neurogenesis in semipalmated sandpipers, their effects however, are seen not during migration, but during the wintering period that follows.

Long distance flight also involves intense exercise, and exercise reliably increases hippocampal neurogenesis [78]. European starlings given flight exercise in a wind tunnel had greater levels of hippocampal neurogenesis than control birds without flight exercise [81]. Starlings flew in the wind tunnel for 15 consecutive days for durations that increased up to 180 min/day, followed by a final day of voluntary flight of up to 4 h. If long distance flight by semipalmated sandpipers causes an increase in hippocampal neurogenesis, however, its effects, like those of cognitive activity and environmental enrichment, are not seen during migration but during the subsequent wintering period.

The same study of starling wind tunnel flight also showed that a diet high in polyunsaturated fatty acids (PUFA) led to less hippocampal neurogenesis than a diet low in PUFAs [81]. Semipalmated sandpipers during their stopover in the Bay of Fundy consume a diet extremely high in PUFAs [2, 82, 83]. Their diet during this stopover includes large amounts of the amphipod *Corophium volutator* in which 45% of total lipids are in the form of PUFAs. This diet may therefore depress hippocampal neurogenesis during stopover.

Stress and elevated glucocorticoid levels reduce hippocampal neurogenesis [43, 44, 46, 47]. The glucocorticoid hormone corticosterone is elevated during long distance migrants in preparation for migration, while accumulating fat reserves for migration, and during refueling stopovers [84–86]. Elevated glucocorticoid levels could therefore be responsible for lower levels of hippocampal neurogenesis found in semipalmated sandpipers collected during their Bay of Fundy stopover.

Finally, there is an alternative interpretation connected to a reduced use of the hippocampus during fall migration. In that case, long distance migration in the sandpipers may rely on compass direction whereas local navigation in the wintering home range may rely on constant use of the hippocampus stimulating neurogenesis in higher proportion. To support this hypothesis, previous findings demonstrated that pigeons with hippocampal ablation usually find their way back home [87]. If similarly fall migration is mainly dependent on compass and less in the HF we may have correspondently less hippocampal neurogenesis in migrating birds.

To find out if DCX-positive cells survived and were integrated into existing hippocampal circuits, or disappear, we checked how neuronal numbers compare in the two groups. The absence of significant difference between migrating and wintering sandpipers with NeuN labeling indicates that hippocampal neuronal number does not increase after each winter, and the newly generated neurons seem to compensate for the loss that might occur during the transatlantic flight. Thus, the difference in hippocampal volumes may be related to other modifications that do not come from neuronal number changes. We suggest that other cell numerical and/or morphological changes and/or expansion of extracellular matrix may contribute to the hippocampal volume differences.

Thus, if long distance migration does act to upregulate neurogenesis its effects are seen not during migration but during the wintering period that follows. Alternatively, there may be diet and glucocorticoid effects that reduce hippocampal neurogenesis during migration. In addition, there may be effects specific to the wintering period that result in elevated neurogenesis, perhaps in preparation for spring migration. Because all these effects and environmental inputs are associated with migration, and because all of them may have an influence on neurogenesis, it would be important in near future to compare more groups of birds at different stages of their annual cycle. Indeed, our study was limited to a group caught in Canada at the beginning of migration and a second group in Brazil during wintering. It would be very informative if there were two more groups of birds, one caught in Canada before migration, when the birds were settled in the region, and one in Brazil just as the return migration started. This design would allow to partially account for dietary, environmental, social (including reproduction) or other factors and stress.

## Conclusions

We hypothesized that neurogenesis in the hippocampal formation of migrating birds would be higher than that of wintering birds. We found higher levels of adult hippocampal neurogenesis and a larger hippocampal formation in wintering birds, suggesting that these changes may be late occurring effects of long distance migratory flight or the result of conditions the birds experienced while wintering. We also detected no differences in NeuN immunolabeled cells in migrating and wintering birds suggesting at least for this time window that neurogenesis just replaced neurons that might be lost during the transatlantic flight. The clear differences we observed between migrating and wintering birds indicate that long distance shorebird migrants provide an opportunity to investigate many questions about the natural control and function of adult hippocampal neurogenesis.

## Supporting information

S1 File Supporting information. Tables A to G.
(DOCX)

S2 File ARRIVE guidelines. ARRIVE guidelines form.
(PDF)
