# Supplementary material for: Hippocampal neurogenesis and volume in migrating and wintering semipalmated sandpipers (Calidris pusilla)
Source: PLoS One. 2017 Jun 7;12(6):e0179134. doi: 10.1371/journal.pone.0179134 (PMC5462419; doi:10.1371/journal.pone.0179134)
Supplement: S1 File — Tables A to G. (DOCX) [file pone.0179134.s001.docx]

**Table A**: **Stereological parameters for the DCX positive neurons in the left hemisphere of Calidris pusilla.**

| **Animal** | **Frame (µm)** | **Grid (µm)** | **N° of Frames** | **N° Sections HF** | **N° Sections T** | **SSF** | **ASF** | **TSF** | **ΣQ‐** |
| --- | --- | --- | --- | --- | --- | --- | --- | --- | --- |
| *C. pusilla* 01 | 140 x 106 | 250 x 250 | 279 | 13 | 22 | 0.167 | 0.237 | 0.427 | 1143 |
| *C. pusilla* 02 | 140 x 106 | 250 x 250 | 234 | 13 | 19 | 0.167 | 0.237 | 0.714 | 1242 |
| *C. pusilla* 03 | 140 x 106 | 250 x 250 | 226 | 14 | 22 | 0.167 | 0.237 | 0.711 | 1275 |
| *C. pusilla* 04 | 140 x 106 | 250 x 250 | 335 | 14 | 17 | 0.167 | 0.237 | 0.605 | 1191 |
| *C. pusilla* 05 | 140 x 106 | 250 x 250 | 316 | 12 | 20 | 0.167 | 0.237 | 0.735 | 2003 |
| *C. pusilla* 06 | 140 x 106 | 250 x 250 | 272 | 10 | 12 | 0.167 | 0.237 | 0.503 | 3025 |
| *C. pusilla* 07 | 140 x 106 | 250 x 250 | 315 | 11 | 15 | 0.167 | 0.237 | 0.620 | 2964 |
| *C. pusilla* 08 | 140 x 106 | 250 x 250 | 308 | 12 | 14 | 0.167 | 0.237 | 0.562 | 3173 |
| *C. pusilla* 09 | 140 x 106 | 250 x 250 | 301 | 11 | 15 | 0.167 | 0.237 | 0.570 | 3176 |
| *C. pusilla* 10 | 140 x 106 | 250 x 250 | 279 | 11 | 13 | 0.167 | 0.237 | 0.570 | 2473 |

ΣQ- = Equals the total number of objects of interest counted using the optical dissector, SSF = Section Sampling Fraction, ASF = Area Sampling Fraction, TSF = Thickness Sampling Fraction, Frame = counting frame size, Grid = grid size. HF= hippocampal formation. T= telencephalon.

**Table B: Stereological parameters for the DCX positive neurons in the right hemisphere of *Calidris pusilla*.**

| **Animal** | **Frame (µm)** | **Grid (µm)** | **N° of Frames** | **N° Sections HF** | **N° Sections T** | **SSF** | **ASF** | **TSF** | **ΣQ‐** |
| --- | --- | --- | --- | --- | --- | --- | --- | --- | --- |
| *C. pusilla* 01 | 140 x 106 | 250 x 250 | 284 | 13 | 22 | 0.167 | 0.237 | 0.427 | 1093 |
| *C. pusilla* 02 | 140 x 106 | 250 x 250 | 233 | 13 | 19 | 0.167 | 0.237 | 0.735 | 1238 |
| *C. pusilla* 03 | 140 x 106 | 250 x 250 | 250 | 14 | 22 | 0.167 | 0.237 | 0.714 | 1406 |
| *C. pusilla* 04 | 140 x 106 | 250 x 250 | 263 | 11 | 17 | 0.167 | 0.237 | 0.633 | 1032 |
| *C. pusilla* 05 | 140 x 106 | 250 x 250 | 321 | 12 | 20 | 0.167 | 0.237 | 0.758 | 2156 |
| *C. pusilla* 06 | 140 x 106 | 250 x 250 | 259 | 10 | 12 | 0.167 | 0.237 | 0.459 | 2709 |
| *C. pusilla* 07 | 140 x 106 | 250 x 250 | 301 | 11 | 15 | 0.167 | 0.237 | 0.573 | 3108 |
| *C. pusilla* 08 | 140 x 106 | 250 x 250 | 284 | 12 | 14 | 0.167 | 0.237 | 0.560 | 2309 |
| *C. pusilla* 09 | 140 x 106 | 250 x 250 | 259 | 11 | 15 | 0.167 | 0.237 | 0.560 | 2698 |
| *C. pusilla* 10 | 140 x 106 | 250 x 250 | 255 | 11 | 13 | 0.167 | 0.237 | 0.549 | 2184 |

ΣQ- = Equals the total number of objects of interest counted using the optical dissector, SSF = Section Sampling Fraction, ASF = Area Sampling Fraction, TSF = Thickness Sampling Fraction, Frame = counting frame size, Grid = grid size. HF= hippocampal formation. T= telencephalon.

| **Animal** | **Frame (µm)** | **Grid (µm)** | **N° of Frames** | **N° Sections HF** | **N° Sections T** | **SSF** | **ASF** | **TSF** | **ΣQ‐** |
| --- | --- | --- | --- | --- | --- | --- | --- | --- | --- |
| *C. pusilla* 01 | 50 x 50 | 250 x 250 | 230 | 15 | 22 | 0.167 | 0.028 | 0.505 | 2294 |
| *C. pusilla* 11 | 50 x 50 | 250 x 250 | 193 | 14 | 19 | 0.167 | 0.028 | 0.332 | 1340 |
| *C. pusilla* 12 | 50 x 50 | 250 x 250 | 235 | 17 | 23 | 0.167 | 0.028 | 0.292 | 1565 |
| *C. pusilla* 03 | 50 x 50 | 250 x 250 | 211 | 19 | 21 | 0.167 | 0.028 | 0.546 | 2345 |
| *C. pusilla* 13 | 50 x 50 | 250 x 250 | 166 | 13 | 22 | 0.167 | 0.028 | 0.317 | 1159 |
| BCCP 01 | 50 x 50 | 350 x 350 | 106 | 09 | - | 0.2 | 0.02 | 0.574 | 1664 |
| BCCP 02 | 50 x 50 | 350 x 350 | 149 | 13 | - | 0.2 | 0.02 | 0.537 | 2135 |
| BCCP 03 | 50 x 50 | 350 x 350 | 177 | 14 | - | 0.2 | 0.02 | 0.556 | 2168 |
| BCCP 04 | 50 x 50 | 350 x 350 | 100 | 10 | - | 0.2 | 0.02 | 0.562 | 2012 |

**Table C: Stereological parameters for the NeuN positive neurons in the left hemisphere of Calidris pusilla.**

ΣQ- = Equals the total number of objects of interest counted using the optical dissector, SSF = Section Sampling Fraction, ASF = Area Sampling Fraction, TSF = Thickness Sampling Fraction, Frame = counting frame size, Grid = grid size. HF= hippocampal formation. T= telencephalon. Animals BCCP 01 to BCCP 04 are from [16].

| **Animal** | **Frame (µm)** | **Grid (µm)** | **N° of Frames** | **N° Sections HF** | **N° Sections T** | **SSF** | **ASF** | **TSF** | **ΣQ‐** |
| --- | --- | --- | --- | --- | --- | --- | --- | --- | --- |
| *C. pusilla* 01 | 50 x 50 | 250 x 250 | 241 | 15 | 22 | 0.167 | 0.028 | 0.485 | 2312 |
| *C. pusilla* 11 | 50 x 50 | 250 x 250 | 218 | 14 | 19 | 0.167 | 0.028 | 0.322 | 1442 |
| *C. pusilla* 12 | 50 x 50 | 250 x 250 | 237 | 17 | 23 | 0.167 | 0.028 | 0.304 | 1641 |
| *C. pusilla* 03 | 50 x 50 | 250 x 250 | 222 | 19 | 21 | 0.167 | 0.028 | 0.549 | 2301 |
| *C. pusilla* 13 | 50 x 50 | 250 x 250 | 173 | 13 | 22 | 0.167 | 0.028 | 0.313 | 1202 |

**Table D: Stereological parameters for the NeuN positive neurons in the right hemisphere of *Calidris pusilla*.**

ΣQ- = Equals the total number of objects of interest counted using the optical dissector, SSF = Section Sampling Fraction, ASF = Area Sampling Fraction, TSF = Thickness Sampling Fraction, Frame = counting frame size, Grid = grid size. HF= hippocampal formation. T= telencephalon.

**Table E. Stereological results of DCX positive Neurons on the left hippocampal formation (LHF) and** **right hippocampal formation (RHF) of migrating and wintering Calidris pusilla**.

SCE = Scheaffer coefficient of error, S.D = Standard deviation, LHF = left hippocampal formation, RHF = right hippocampal formation.

| **Migrating** | **Capture Date** | **N° of DCX LHF** | **SCE LHF** | **Thickness**  **(µm) LHF** | **DCX/mm³**  **LHF** | **N° of DCX RHF** | **SCE RHF** | **Thickness (µm) RHF** | **DCX/mm^3^**  **RHF** |
| --- | --- | --- | --- | --- | --- | --- | --- | --- | --- |
| *C.pusilla* 01 | 04/08/2012 | 67,541.45 | 0.034 | 23.4 | 16,081.30 | 64,694.47 | 0.033 | 23.3 | 14,376.55 |
| *C.pusilla* 02 | 04/08/2012 | 43,855.88 | 0.045 | 20.4 | 12,182.19 | 42,555.52 | 0.043 | 20.3 | 11,820.98 |
| *C.pusilla* 03 | 12/08/2012 | 45,299.09 | 0.047 | 22.0 | 12,942.60 | 49,850.47 | 0.046 | 20.6 | 13,473.10 |
| *C.pusilla* 04 | 07/08/2012 | 49,807.38 | 0.041 | 24.4 | 9,223.59 | 41,232.15 | 0.047 | 23.3 | 9,817.18 |
| *C.pusilla* 05 | 07/08/2012 | 68,785.96 | 0.048 | 20.5 | 13,487.44 | 71,830.84 | 0.044 | 19.5 | 14,084.48 |
| Mean |  | 55,057.95 | 0.043 | 21.7 | 12,783.42 | 54,032.69 | 0.040 | 21.4 | 12,714.46 |
| S.D. |  | 12,171.50 | 0.006 | 2.0 | 2,471.38 | 13,633.76 | 0.006 | 1.78 | 1,897.87 |
| **Wintering** | **Capture Date** | **Nº of DCX LHF** | **SCE LHF** | **Thickness (µm) LHF** | **DCX/mm³**  **LHF** | **Nº of DCX RHF** | **SCE RHF** | **Thickness (µm) RHF** | **DCX/mm^3^**  **RHF** |
| *C.pusilla* 06 | 03/03/2009 | 151,874.95 | 0.048 | 25.7 | 26,185.3 | 149,374.03 | 0.046 | 31.0 | 27,158.91 |
| *C.pusilla* 07 | 14/01/2014 | 142,769.19 | 0.040 | 28.9 | 20,395.6 | 140,220.59 | 0.039 | 26.3 | 21,572.40 |
| *C.pusilla* 08 | 10/11/2014 | 120,850.48 | 0.038 | 26.6 | 18,310.7 | 101,864.27 | 0.044 | 25.3 | 17,870.92 |
| *C.pusilla* 09 | 10/11/2014 | 140,695.00 | 0.036 | 26.1 | 22,332.5 | 121,964.11 | 0.044 | 26.4 | 22,585.95 |
| *C.pusilla* 10 | 10/11/2014 | 109,529.36 | 0.037 | 26.6 | 17,955.6 | 100,330.13 | 0.047 | 26.2 | 18,930.21 |
| Mean |  | 133,143.80 | 0.040 | 26.1 | 21,035.96 | 122,750.63 | 0.040 | 27.0 | 21,623.68 |
| S.D. |  | 15,551.80 | 0.005 | 1.9 | 3,373.53 | 22,099.45 | 0.003 | 2.26 | 3,636.80 |

| **Migrating** | **Capture Date** | **Estimated Vol. (mm³) LHF** | **CE**  **Gundersen**  **m=1 LHF** | **Estimated Vol. (mm³) LT** | **CE**  **Gundersen**  **m=1 LT** | **Vol. LHF**  **/Vol. LT** | **Estimated Vol. (mm³) RHF** | **CE Gundersen**  **m=1 RHF** | **Estimated Vol. (mm³) RT** | **CE Gundersenm=1 RT** | **Vol. RHF**  **/Vol. RT** |
| --- | --- | --- | --- | --- | --- | --- | --- | --- | --- | --- | --- |
| *C.pusilla* 01 | 04/08/2012 | 5.180 | 0.022 | 84.60 | 0.008 | 0.061 | 5.31 | 0.022 | 89.1 | 0.006 | 0.060 |
| *C.pusilla* 02 | 04/08/2012 | 3.580 | 0.030 | 80.40 | 0.009 | 0.044 | 3.56 | 0.030 | 77.3 | 0.008 | 0.046 |
| *C.pusilla* 03 | 12/08/2012 | 3.870 | 0.026 | 82.70 | 0.007 | 0.046 | 3.78 | 0.027 | 71.4 | 0.008 | 0.053 |
| *C.pusilla* 04 | 07/08/2012 | 4.610 | 0.022 | 88.90 | 0.007 | 0.051 | 5.15 | 0.021 | 79.6 | 0.006 | 0.065 |
| *C.pusilla* 05 | 07/08/2012 | 5.100 | 0.020 | 105.0 | 0.005 | 0.048 | 5.10 | 0.019 | 97.9 | 0.004 | 0.052 |
| Mean |  | 4.468 | 0.024 | 88.32 | 0.007 | 0,050 | 4.58 | 0.024 | 83.0 | 0.006 | 0.055 |
| S.D. |  | 0.7 | 0.004 | 9.83 | 0.001 | 0.002 | 0.84 | 0.005 | 10.4 | 0.006 | 0.007 |
| **Wintering** | **Capture**  **Date** | **Estimated Vol. (mm³) LHF** | **CE Gundersen**  **m=1 LHF** | **Estimated Vol. (mm³) LT** | **CE Gundersen m=1 LT** | **Vol. LHF**  **/Vol. LT** | **Estimated Vol. (mm³) RHF** | **CE Gundersen**  **m=1 RHF** | **Estimated Vol. (mm³) RT** | **CE Gundersenm=1 RT** | **Vol. RHF**  **/Vol. RT** |
| *C.pusilla* 06 | 03/03/2009 | 5.82 | 0.023 | 67.70 | 0.023 | 0.085 | 5.55 | 0.024 | 72.4 | 0.020 | 0.077 |
| *C.pusilla* 07 | 14/01/2014 | 6.66 | 0.022 | 108.0 | 0.008 | 0.061 | 6.39 | 0.019 | 111.0 | 0.007 | 0.058 |
| *C.pusilla* 08 | 10/11/2014 | 6.45 | 0.024 | 95.30 | 0.005 | 0.067 | 5.28 | 0.025 | 99.2 | 0.006 | 0.053 |
| *C.pusilla* 09 | 10/11/2014 | 6.24 | 0.026 | 117.0 | 0.006 | 0.053 | 5.37 | 0.029 | 120.0 | 0.006 | 0.045 |
| *C.pusilla* 10 | 10/11/2014 | 6.27 | 0.023 | 81.90 | 0.011 | 0.076 | 5.25 | 0.025 | 83.1 | 0.011 | 0.063 |
| Mean |  | 6.288 | 0.024 | 93.98 | 0.011 | 0.069 | 5.57 | 0.024 | 97.1 | 0.010 | 0.059 |
| S.D. |  | 0.31 | 0.002 | 19.8 | 0.007 | 0.01 | 0.47 | 0.024 | 19.5 | 0.020 | 0.012 |

**Table F. The volume estimates for the hippocampal formation, telencephalon, and the ratio between them for migrating and wintering *Calidris pusilla.***

CE=Coefficient of error, LT= left telencephalon, LHT= Left hippocampal formation, RT=right telencephalon, RHF= Right hippocampal formation, Vol.=Volume.

**Table G. The NeuN positive neurons on hippocampal formation of Calidris pusilla.**

| **Migrating** | **Capture Date** | **Nº of NeuN**  **LHF** | **SCE LHF** | **Thickness (µm) LHF** | **NeuN/mm^3^**  **LHF** | **Nº of NeuN RHF** | **SCE RHF** | **Thickness (µm) RHF** | **NeuN/mm^3^ RHF** |
| --- | --- | --- | --- | --- | --- | --- | --- | --- | --- |
| *C. pusilla* 01 | 04/08/2012 | 982,519.38 | 0.042 | 19.0 | 189,675.56 | 1,030,756.2 | 0.04 | 20.1 | 194,116.05 |
| *C. pusilla* 11 | 04/08/2012 | 871,772.44 | 0.039 | 30.0 | 243,511.85 | 968,726.25 | 0.038 | 31.0 | 272,114.12 |
| *C. pusilla* 12 | 07/08/2012 | 1,160,225.63 | 0.036 | 33.0 | 299,799.90 | 1,165,632.5 | 0.034 | 32.1 | 308,368.39 |
| *C. pusilla* 03 | 07/08/2012 | 927,566.44 | 0.040 | 18.0 | 201,207.47 | 905,189.75 | 0.036 | 18.3 | 175,765.00 |
| *C. pusilla* 13 | 06/08/2012 | 789,153.00 | 0.036 | 31,0 | 154,735.88 | 832,040.50 | 0.039 | 31.6 | 163,145.20 |
| Mean |  | 946,247.37 | 0.040 | 26.2 | 217,786.13 | 980,469.05 | 0.040 | 26.6 | 222,701.75 |
| S.D. |  | 139,352.5 | 0.003 | 7.1 | 55,741.51 | 127,132.02 | 0.004 | 6.81 | 63,928.75 |
| **Wintering** | **Capture Date** | **Nº of NeuN**  **LHF** | **SCE LHF** | **Thickness (µm) LHF** | **NeuN/mm^3^**  **LHF** | **Nº of NeuN RHF** | **SCE RHF** | **Thickness (µm)** | **NeuN/mm^3^** |
| BCCP 01 | 18/01/2012 | 723,297 | 0.04 | 17.7 | - | - | - | - | - |
| BCCP 02 | 04/02/2012 | 1,040,384 | 0.04 | 19.3 | - | - | - | - | - |
| BCCP 03 | 04/02/2012 | 982,961 | 0.04 | 18.3 | - | - | - | - | - |
| BCCP 04 | 04/12/2012 | 891,518 | 0.04 | 18.4 | - | - | - | - | - |
| Mean |  | 909,504 | 0.04 | 18.41 | - | - | - | - | - |
| S.D. |  | 138,470.27 | 0.0022 | 0.7 | **-** | **-** | **-** | **-** | **-** |

SCE = Scheaffer coefficient of error, S.D = Standard deviation, LHF = left hippocampal formation, RHF = right hippocampal formation. Animals BCCP 01 to BCCP 04 are from [16].
